# Advances and Challenges in Diagnosis and Management of Heart Failure

**DOI:** 10.3390/diagnostics12051103

**Published:** 2022-04-28

**Authors:** Ryan D. Sullivan, Inna P. Gladysheva

**Affiliations:** Department of Medicine, University of Arizona College of Medicine—Phoenix, Phoenix, AZ 85004, USA

The prevalence of heart failure (HF) with reduced (r) and preserved (p) ejection fraction (EF) continues to rise globally despite current advances in diagnostics and improvements to medical management. Regardless of the underlying etiology, HF remains a progressive disease, which is largely irreversible and may ultimately require cardiac transplantation. This Special Issue focuses on the challenges and recent advances of diagnosis, treatment, and prevention of HF with or without associated comorbidities.

Existing clinical imaging modalities and methods continue to be optimized for HF. These advances allow for earlier initial diagnosis, improved prognostic timelines, and can even be used to provide personalized medical management. Echocardiography remains a clinical mainstay for point-of-care evaluation, identifying disease etiology, and longitudinal monitoring. From our issue, Pescariu, et al. utilized cardiac strain and left ventricular function to accurately predict resynchronization success in HFrEF patients [1]. Left ventricular end-diastolic diameter, mitral valve E/e’ ratio, and left ventricular outflow tract velocity time integral were reported by Zamfirescu, et al. to predict HF readmission following initial acute HFpEF [2]. Masarone et al. reviewed how echocardiography is utilized to monitor graft function, pathology development and rejection following heart transplantation [3]. The ability to measure standardized views, while simultaneously having the versatility to explore new correlates has clearly earned cardiac ultrasound a top spot in HF patient evaluation and management.

Blood biomarkers are another well-established component for helping in the diagnosis of HF. While the natriuretic peptides (BNP/NT-proBNP and ANP/NT-proANP) are the most commonly used clinical biomarkers to confirm or exclude a HF diagnosis, other biomarkers are under investigation, Though there were no correlations with serum galectin-3 or copeptin, Ianos, et al. found fibroblast growth factor 21 (FGF21) to be a reliable biomarker for HFpEF with type-2 diabetes mellitus [4]. Considering acute myocardial infarction (AMI) as a risk for developing HF, Tilea et al. reviewed biomarkers in the pathophysiology that are altered earlier than myocyte necrosis needed to release cardiac troponins [5] which are commonly assessed during emergency workups. By combining modalities, echocardiography and blood biomarkers, clinicians can more confidently diagnose HF and likely stage the disease severity. Pecherina et al. found using both modalities (diastolic dysfunction + NT-proBNP, sST2, galectin-3, and MMP-3) can predict risk for adverse cardiac remodeling in patients with HFpEF following ST-segment elevation myocardial infarction (STEMI) [6]. Similarly in both HFrEF and HFpEF STEMI patients, Oleynikov et al. used acute left ventricular remodeling (ALVR vs. non-ALVR) status to predict and stratify outcome risks with statistical significance [7].

Even with proper imaging and great blood biomarkers, common comorbidities (diabetes mellitus, COPD, chronic kidney disease, etc.) can complicate accurate and timely diagnosis of HF. Lai et al. found that non-alcoholic fatty liver disease without HF is associated with left ventricular diastolic dysfunction and subclinical changes in left atrial contractility [8]. Adamska-Welnicka et al. reviewed the difficulty associated with proper HF diagnosis when comorbidities of chronic kidney disease or pulmonary hypertension are present [9].

Part of the confusion from the comorbidities is the non-cardiac-systemic involvement; notably, the lungs. Ventilatory inefficiency, measured during cardiopulmonary exercise test, was used to stratify left ventricular ejection fraction in HFrEF outpatients for prognostic predictability of HF outcomes [10]. Chen et al. reiterated that ventilatory inefficiency (due to tachypnea) can in part be due to receptors responding to pulmonary congestion [10]. Early detection of pulmonary edema and congestion responsible for HF decompensation is lacking clinically, Pirrotta et al. reviewed traditional methods and chest radiography vs. lung ultrasonography (LUS) [11] as an alternative method to access decompensation. The standardization of LUS in HF evaluations would improve outcomes by diagnosing the initiation of lung fluid rather than relying on peripheral edema (advanced) or other HF symptoms to begin diuretic treatment. Edema, regardless of anatomical location, is synonymous with clinical symptoms and late stage HF. Thankfully a new drug class reviewed by Hernandez et al., sodium-glucose cotransporter-2 inhibitors (SGLT-2i), are helping to delay HFrEF progression, rehospitalization and improve quality of life by reducing HF associated edema [12].

HF medical management effectiveness can be longitudinally evaluated using all the above methods. This improvement in patient monitoring allows for a personized medicine approach–run the diagnostics and alter therapy according to the patient’s own data. As an example, Oleynikov, et al. were able to generate patient risk stratification using a developed model (formula) to predict chronic HF progression within 48 weeks after STEMI [13]. Though not as effective in cardiomyopathy of ischemic origin, Poglajen et al. found that the addition of angiotensin receptor blocker–neprilysin inhibitor (ARNI) therapy improved left and right ventricular function beyond that of control patients on optimal medical treatment after one year [14].

We thank the authors, reviewers, Ms Lassie Shu and the rest of the editorial staff that contributed to this Special Issue. We hope that readers will appreciate the wide breadth of topics and clinical utility of the articles and reviews published within “Diagnosis and Management of Heart Failure”.
